# Application of an Electronic Nose to the Prediction of Odorant Series in Wines Obtained with *Saccharomyces* or Non-*Saccharomyces* Yeast Strains

**DOI:** 10.3390/molecules30071584

**Published:** 2025-04-02

**Authors:** Raquel Muñoz-Castells, Margherita Modesti, Jaime Moreno-García, Alexandro Catini, Rosamaria Capuano, Corrado Di Natale, Andrea Bellincontro, Juan Moreno

**Affiliations:** 1 Department of Agricultural Chemistry, Edaphology and Microbiology, Agrifood Campus of International Excellence CeiA3, University of Córdoba, Marie Curie (C3) and Severo Ochoa (C6) Buildings, Ctra. N-IV-A, km 396, 14014 Córdoba, Spain; b52mucar@uco.es (R.M.-C.); b62mogaj@uco.es (J.M.-G.); 2 Department for Innovation of Biological, Agrofood and Forest Systems (DIBAF), University of Tuscia, 01100 Viterbo, Italy; bellin@unitus.it; 3 Department of Electronic Engineering, University of Rome Tor Vergata, Via del Politecnico 1, 00133 Rome, Italy; catini@ing.uniroma2.it (A.C.); capuano@ing.uniroma2.it (R.C.); dinatale@uniroma2.it (C.D.N.)

**Keywords:** active dry yeast, electronic nose, chemometric analysis, odor activity values, PLS-DA

## Abstract

Electronic noses (E-noses) have become powerful tools for the rapid and cost-effective differentiation of wines, providing valuable information for the comprehensive evaluation of aroma patterns. However, they need to be trained and validated using classical analytical techniques, such as gas chromatography coupled with mass spectrometry, which accurately identify the volatile compounds in wine. In this study, five low-ethanol wines with distinctive sensory profiles—produced using *Saccharomyces* and non-*Saccharomyces* yeasts and tailored to modern consumer preferences—were analyzed to validate the E-nose. A total of 57 volatile compounds were quantified, 27 of which had an Odor Activity Value (OAV) over 0.2. The content in volatiles, grouped into 11 odorant series according to their odor descriptors, along with the data provided by 12 E-nose sensors, underwent advanced statistical treatments to identify relationships between both data matrices. Partial least squares discriminant analysis (PLS-DA) applied to the data from the 12 E-nose sensors revealed well-defined clustering patterns and produced a model that explained approximately 92% of the observed variability. In addition, a principal component regression (PCR) model was developed to assess the ability of the E-nose to non-destructively predict odorant series in wine. The synergy between the volatile compound profiles and the pattern recognition capability of the E-nose, as captured by PLS-DA, enables a detailed characterization of wine aromas. In addition, predictive models that integrate data from gas chromatography, flame ionization detection, and mass spectrometry (GC-FID/GC-MSD) with the electronic nose demonstrating a promising approach for a rapid and accurate odor series prediction, thereby increasing the efficiency of wine aroma analysis.

## 1. Introduction

Today, the market offers a wide variety of wines whose sensory profile is the main distinguishing feature, and one of the main challenges for modern enology is to control the aroma profile of its wines to meet consumer expectations. Among the hundreds of volatile molecules involved in wine aroma properties, those of fermentative origin produced by the secondary metabolism of yeast have been identified as the most important contributors.

Yeasts are single-celled microorganisms that are essential for the production of fermented foods such as bread, beer, and wine. In winemaking, they convert grape sugars into alcohol, carbon dioxide, and secondary metabolites. *Saccharomyces cerevisiae* is the most commonly used species for fermentation and certain strains of this species have a significant impact on wine aroma, flavor, and quality [1,2,3]. For this reason, yeast strain selection has become increasingly important to meet consumer demands and to mitigate the adverse effects of climate change, which negatively affect the fermentation behavior and sensory quality of wines [4]. To overcome these challenges, the use of active dry yeast (ADY) strains has become increasingly popular. ADY helps prevent sluggish or stuck fermentations, ensuring a more consistent process and delivering distinct sensory characteristics compared to spontaneous fermentation [5,6]. In addition, the use of selected ADY strains to enhance metabolite production, combined with alternative fermentation strategies, such as sequential fermentations and yeast immobilization [7], are valuable biotechnological tools helping to minimize the impact of climate change [5,8] and to obtain new wine types.

Commercial yeast strains, both *Saccharomyces* and non-*Saccharomyces*, are available, with the former being particularly important in the wine industry, as noted above [9]. However, non-*Saccharomyces* yeasts have recently gained popularity for producing wines with desirable flavors and complex aromas. These yeasts release primary aromas from grape precursors through a glycosidase enzyme-driven hydrolysis mechanism [10]. Among the most recommended non-*Saccharomyces* yeasts, *Lachancea thermotolerans* improves wine acidity and *Metschnikowia pulcherrima* improves the microbial stability of wines [7,11,12]. Proper fermentation management directly affects the aroma compounds that shape the chemical and sensory complexity of wine. As a result, the objective characterization of wine aroma remains challenging due to its complex and variable composition in volatile molecules. Because of this complexity, human evaluation is still the most common method for assessing wine aroma quality, despite its inherent subjectivity. This makes the development of an objective, reproducible, and user-friendly analytical method an important research focus for both consumers and the wine industry.

Electronic noses (E-noses) are innovative tools equipped with various sensors designed to detect changes in volatile compounds in complex matrices. They have applications in multiple research fields, including the wine industry, where they help analyze qualitative differences in wine volatile compounds in their headspace. Specifically, E-noses can identify the wine’s aging process [13], determine the winemaking methodology used [14], establish its geographical origin, and predict certain sensory attributes [15]. Additionally, they are valuable for detecting off-flavors and other sensory defects, helping wine quality classification [16,17]. In recent years, E-noses have been increasingly used in wine research due to their ability to provide a volatile fingerprint of wines, enabling easy differentiation and authenticity verification. Overall, E-noses provide a cost-effective alternative to traditional methods, delivering rapid and reliable analyses while reducing the need for sensory panels and expensive chemical techniques [18].

One of the key advantages of E-nose technology is its cost-effectiveness compared to traditional methods. Maintaining a sensory panel requires ongoing investment in organization, training, and upkeep [19]. Recruiting trained panelists and conducting regular taste sessions can be a significant financial burden, especially for small- and medium-sized wineries. Additionally, sensory panel testing is time-consuming and limited by the number of samples that can be analyzed in a single session due to human sensory fatigue [20]. Highly trained sensory experts develop exceptional sensory acuity through rigorous, standardized training. However, their evaluations are still influenced by individual sensory thresholds, genetic variability, cultural background, and experience [21]. The variability in perception among tasters—even experts—is not simply “subjectivity” in the casual sense but rather a natural consequence of human sensory diversity and cognitive factors [21]. Research suggests that individual sensory responsiveness, memory, and emotional reactions contribute to differences in perception, even when rigorous training minimizes variability [22,23]. These differences highlight the importance of complementary technologies, such as E-noses, which provide consistent, reproducible, and objective data on volatile profiles without the cognitive biases inherent in human tasters. On the other hand, analytical instruments, such as GC-MS technologies, while providing precise and objective characterization, require significant capital investment for equipment, maintenance, and highly specialized personnel. The time required for sample preparation, analysis, and data interpretation can also slow decision-making in production and quality control [24]. In contrast, E-nose devices offer a rapid, non-destructive, and user-friendly alternative. Once calibrated and validated, an E-nose system can deliver real-time data with minimal operator input, enabling faster decision-making [25,26].

This study focuses on predictive models that integrate quantitative data from traditional analytical methods with data from an E-nose equipped with 12 quartz microbalance (QMB) sensors. The goal is to assess whether this non-destructive method can effectively distinguish wine aroma compositions. The analysis relies on Odor Activity Values (OAVs) of 11 odorant series, which categorize all quantified volatiles based on their odor descriptors and are calculated by summing the OAVs of individual compounds. This study provides new insights into using E-nose technology to quantify aroma profiles in wines fermented from the same grape must, comparing *Saccharomyces* and non-*Saccharomyces* yeasts in both suspended and immobilized forms.

## 2. Results and Discussion

### 2.1. Enological Parameters and Fermentation Dynamics

Five young white wines obtained from the same grape must, but fermented with five different strategies, have been analyzed using both traditional techniques and an E-nose equipped with 12 quartz microbalances, as described in Section 3.4. Briefly, the fermentation and their corresponding wines were labelled as follows: spontaneous fermentation (WY) and four inoculation methods—*Saccharomyces cerevisiae* active dry yeast (SC); *M. pulcherrima* active dry yeast (MP); *L. thermotolerans* yeast in free format (LT); and *L. thermotolerans* encapsulated in biocapsules (BCs).

The initial must had a density of 1088 g L^−1^ (about 208 g L^−1^ sugars), which decreased to 990 g L^−1^ after 11 days of fermentation. The enological parameters were analyzed, and the values are presented in Table 1. All wines contained residual sugar levels below 1 g L^−1^, while ethanol concentrations ranged from 12.3 ± 0.3% to 14.1 ± 0.01% (*v*/*v*), with *L. thermotolerans* yielding the lowest levels. This is particularly relevant for mitigating the effects of climate change, as rising global temperatures lead to increased grape sugar concentrations, which in turn result in higher ethanol levels in wine. In this context, whereas ethanol reduction via *S. cerevisiae* strains requires complex genetic modifications, the use of *L. thermotolerans* offers a simpler and more effective alternative [8].

The total acidity ranged from 6.3 to 7.7 g L^−1^ in the SC, MP, and WY wines, but was significantly higher in LT and BC, reaching 9.5 and 8.8 g L^−1^, respectively. All wines exhibited volatile acidity values within the acceptable range for the young white wines, which should not exceed 0.5 g L^−1^. Lactic acid concentrations were low in all samples except for the LT and BC wines, where levels ranged between 4.0 and 5.0 g L^−1^. Conversely, malic acid content was lowest in *L. thermotolerans* wines, whereas WY wines exhibited the highest content. Although SC wines were expected to have the highest glutathione levels (approximately 2 mg L^−1^), LT wines surpassed them, reaching over 6 mg L^−1^. Glutathione is a tripeptide made of glutamate, cysteine, and glycine. It acts as a key antioxidant, protecting cells from oxidative stress by neutralizing free radicals. These results align with findings by Vicente et al. (2021) [27] and Binati et al. (2022) [28], who reported glutathione production by certain non-*Saccharomyces* yeasts.

### 2.2. Key Volatile Compounds

Table 2 presents the OAV values for the 57 quantified volatile compounds, calculated as the ratio of the concentration to odor perception threshold (OPT). It also displays the homogeneous groups (HGs) determined for each compound at a significance level of *p* ≤ 0.05. These volatiles belong to several chemical families, including acetates, ethyl esters, higher alcohols, lactones, carbonyl compounds, and terpenes. Their CAS numbers, OPT values, odor descriptors, and corresponding odorant series are provided in Supplementary Material Table S1. Among the quantified volatiles, 27 exhibited an OAV greater than 0.2 in certain wines, suggesting a significant impact on their aroma [29,30]. These values for each of the wines allow an approximation of the volatiles to the global wine aroma of the wine, which is used as a common methodology in research [31,32]. In this regard, it is well established that yeasts play a determinant role in the synthesis of aroma compounds as by-products of their metabolic activity during alcoholic fermentation [33]. Some of these compounds are mainly produced by the non-*Saccharomyces* yeasts and give special and distinctive characteristics to the wine aroma due to their different capacities to synthesize metabolites compared to *Saccharomyces* [8,34].

Compounds such as acetaldehyde, ethyl acetate, 3-methyl-1-butanol, and 2-phenylethanol, among the major volatiles, have the highest OAVs. Apart from yeasts, other factors such as fermentation conditions or assimilable nitrogen can influence the production of these compounds [35]. In addition, the odorants among the minor volatiles, including isoamyl acetate, 2-phenylethyl acetate, cis-3-hexenyl butyrate, β-damascenone, and limonene, also show high OAVs. Esters of acetic acid with higher alcohols impart fruity and floral aromas to the wine. In general, wines obtained with non-*Saccharomyces* yeasts have higher OAVs for ethyl acetate, while the acetates of 2-phenethanol and isoamyl alcohol acetates are higher in wines from *Saccharomyces* yeasts. The latter two compounds enhance floral and fruity aromas by adding complexity to the overall wine aroma [8]. Wines from *M. pulcherrima* and *L. thermotolerans* are characterized by the production of higher alcohols, as recently reported by other authors [8]. However, it is known that higher alcohol contents below 400 mg L^−1^ contribute positively to the wine aroma and flavor, but the aroma quality decreases when this level is exceeded [7,12].

The concentrations of the esters isoamyl acetate, 2-phenethyl acetate, ethyl methyl butyrate, ethyl decanoate, ethyl dodecanoate, and ethyl tetradecanoate exhibited a significant increase in their OAVs in wines fermented with *L. thermotolerans* immobilized in biocapsules. This increase may be attributed to the method of introducing the starter culture into the must and align with findings reported by other researchers [7,36]. Regarding the terpene family, limonene exhibits OAV values of 468 in WY wines and 320 in BC wines, making it a key aroma compound. Initially detected in aromatic grape varieties such as Muscat, limonene is released by non-*Saccharomyces* yeasts through the hydrolysis of glycosidic bonds. This process is due to their β-glycosidase enzyme system, which cleaves glycosidic precursors to release free volatile terpenes [37]. Additionally, limonene can originate from the dehydroxylation and cyclization of nerol, a transformation catalyzed by enzymatic activity during fermentation or induced by the low pH conditions in wine [38]. This reaction imparts a fresh citrus aroma reminiscent of orange [39].

### 2.3. Odorant Series Values and E-Nose Data Matrices

**Figure 1:**
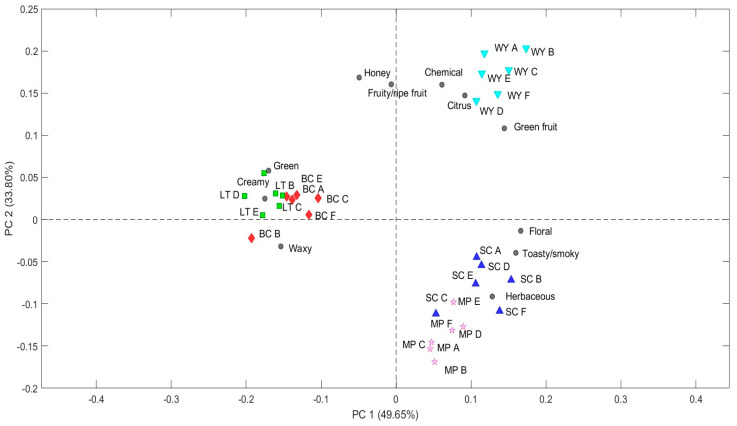
Biplot (PC1 vs. PC2) of the PCA performed on auto-scaled odorant series’ measurement. The odorant data were used as predictor variables, while the wines produced with the different yeast strains (WY: wild type; SC: *Saccharomyces cerevisiae*; MP: *Metschnikowia pulcherrima*; LT: *Lachancea thermotolerans*; and BC: biocapsules of *L. thermotolerans*) were used as response variables.

The 11 odorant series (OS) selected in accordance with Muñoz-Castells et al. (2023) [12] include the following categories: chemical, fruity/ripe fruit, green fruit, green, floral, creamy, citrus, herbaceous, toasty/smoky, honey, and waxy. The OAVs for each series were calculated as the sum of the OAVs of volatile compounds assigned to each category (Table S2). This data matrix was then subjected to a PCA and three principal components (PCs) were selected, explaining a cumulative variance of 94.22% (PC1: 49.65%, PC2: 33.80%, and PC3: 10.77%). The resulting biplot (Figure 1) reveals three different clusters. The first cluster corresponds to the wines from spontaneous fermentation (WY), characterized by citrus, chemical, green fruit, honey, and fruity aromas. The second cluster groups *S. cerevisiae* (SC) and *M. pulcherrima* (MP) wines, exhibit floral, herbaceous, and toasty/smoky notes. The third cluster consists of wines fermented with *L. thermotolerans* (both immobilized and free formats), characterized by creamy, green, and waxy aromas.

The *S. cerevisiae* and non-*Saccharomyces* yeast strains tested in this study produced wines with different aroma profiles due to their specific metabolic pathways and enzymatic activities. These results are consistent with previous studies demonstrating the influence of the interaction between grape-derived compounds and yeast metabolism on wine aroma [40]. *L. thermotolerans*, both in its free and immobilized form, forms a single cluster, as several studies have shown that wines produced with both formats have similar sensory profiles, with no significant differences in several of the volatile compounds analyzed [41,42].

The application of PCA to the OS data matrix effectively highlights these differences, providing a chemical basis for clustering wines based on their volatile compound content. On the other hand, the E-nose used in this research evaluates aroma by detecting changes in the oscillation frequency of its 12 QMB sensors, which occurs due to the adsorption of volatile compounds [18]. These signals are then processed using several statistical approaches to identify clusters corresponding to the wines analyzed. The database compiled with OS and E-nose data serves as a valuable resource for establishing relationships between these two methodologies, opening the way for an objective, simple, and fast approach to identify differences in wine aroma profiles.

**Figure 2:**
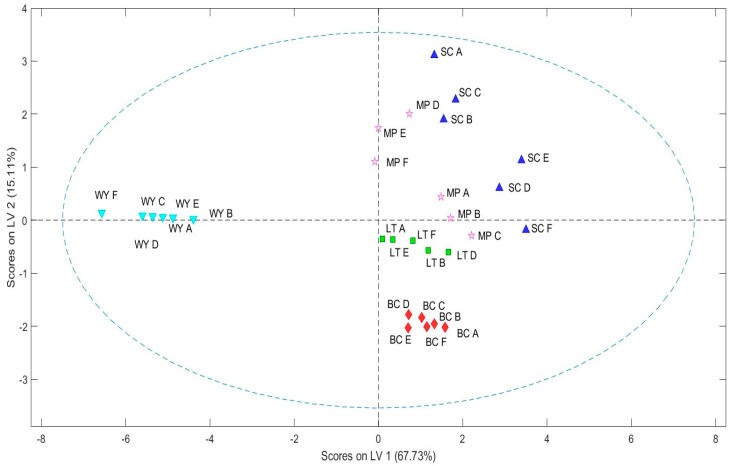
Score plot (LV1 vs. LV2) of the PLS-DA performed on auto-scaled E-nose measurement. The responses of the QMBs were used as predictor variables while the wines produced with the different yeast strains (WY: obtained by spontaneous fermentation; SC: using *Saccharomyces cerevisiae* active dry yeast; MP: using *Metschnikowia pulcherrima* active dry yeast; LT: using *Lachancea thermotolerans* yeast in free format; and BC: with *L. thermotolerans* immobilized) were used as response variables.

**Figure 3:**
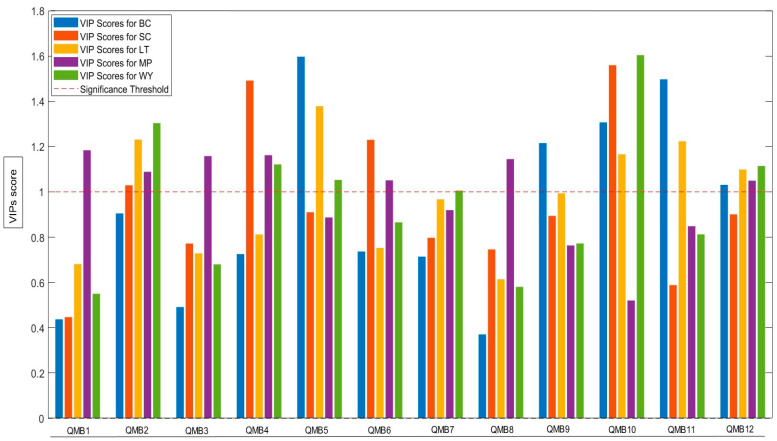
Variables of importance in projection (VIPs) generated by each QMB with an efficacy threshold base equal to one. The plot shows the effect of the variables [loadings and QMBs] on the pattern recognition discrimination of the partial least squares discriminant analysis.

The signal data collected from the 12-sensor array of the E-nose were subjected to PLS-DA and transformed into new variables through a linear combination procedure. Figure 2 and Figure 3 show the PLS-DA score plot and the corresponding variables of importance in projection (VIPs) plot, illustrating the influence of the 12 sensors on the selected latent variables. The resulting model explains 92.25% of the total variance within the first three latent variables (LV1: 67.73%, LV2: 15.11%, and LV3: 9.41%). The score plot (Figure 2) reveals two main clusters: The first comprises wines produced through spontaneous fermentation with wild yeast (WY), which are clearly separated from the other group. The second cluster corresponds to wines obtained with different commercial yeast strains. This second cluster can be further subdivided into two additional clusters: one containing wines from *S. cerevisiae* (SC) or *M. pulcherrima* (MP), and another that includes wines from *L. thermotolerans* inoculated either freely or in biocapsule format (LT and BC).

Both the PCA model (based on OS data) and the PLS-DA model (from E-nose data) reveal consistent clustering patterns. For instance, spontaneous fermentation (WY) forms a unique cluster in both analyses, strongly characterized by citrus, green fruit, and honey aromas. As suggested by other authors, spontaneously fermented wines show differences in their aromatic profile compared to commercial yeast fermentations [6,43,44]. These differences are due to the complexity of the indigenous microorganisms [44], although differences between cluster groups are also attributed to variations in the intensity of common compounds [6]. Likewise, wines from SC and MP or those from LT group together based on shared odorant series (e.g., floral and toasty for SC and MP; and creamy and waxy series for LT). These results highlight the strong relationships between volatile-based chemical characterization using OS and the pattern recognition capabilities of the E-nose in wine aroma profiling.

Figure 3 illustrates the variables of importance in projection (VIPs) that drive the segregation of the sample scores. Notably, all QMB sensors have VIP values greater than one, with the only exception being QMB7. This suggests that most sensors contribute to the clustering of the five wines tested. The performance of the PLS-DA model is further evaluated using the confusion matrix (Supplementary Material, Table S3). During calibration, all five wines tested were identified with 100% accuracy. Cross-validation also maintained reasonably high accuracy, although some misclassifications occurred for BC, LT, and SC.

**Figure 4:**
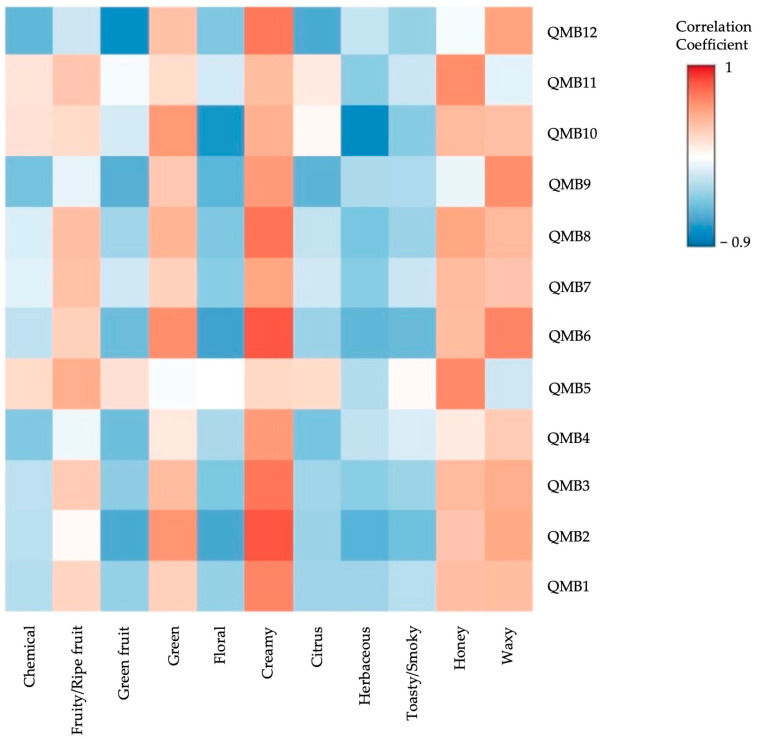
Heatmap illustrating the Pearson correlation analysis between the odorant series (horizontal axis) and E-nose sensor signals (QMB1 to QMB12 on the vertical axis). Correlation values are represented by a color gradient, with red shades indicating strong positive correlations and blue shades indicating strong negative correlations, as shown on the correlation coefficient scale on the right.

To further explore the correlation between the OS and E-nose sensor signals, a Pearson correlation analysis was conducted on these datasets (Figure 4). The “chemical” series showed a positive correlation with the QMB sensors 5, 10, and 11. This series strongly characterized the wild yeast (WY) wines, and as expected, the VIP plot shows high scores for these sensors in WY wines. Similarly, the “fruity/ripe fruit” series was positively correlated exclusively with the QMB sensors 5 and 4, which also have the highest VIP values for WY, consistent with the PCA observations of the OS data.

In contrast, the green series characterizing LT and BC wines was positively correlated with all sensors except QMB 5. Conversely, series such as “floral”, “citrus”, “herbaceous”, and “toasty/smoky” showed negative correlations with all sensors. On the other hand, series such as “creamy”, “honey”, and “waxy” were positively correlated with most QMB sensors, with only a few exceptions. As other authors have noted, electronic noses have a greater ability to detect and correlate wine compounds without being affected by human variability. In contrast, sensory analysis, although linked to real aroma perception, is influenced by natural differences between individuals [45].

Lastly, to verify the ability of E-nose sensors to predict OS in wine samples, a principal component regression analysis (PCR) was performed. PCR is a statistical technique used to model the relationships between dependent variables (E-nose data) and independent variables (OS data), particularly in cases of strong correlations and multicollinearity. This method provides insight into the interrelationships between variables, as previously demonstrated in studies evaluating the predictive ability of the E-nose for individual volatile compounds [14]. For the construction of the PCR model, five principal components were selected, explaining 98.85% of the cumulative variability (PC1: 67.83%, PC2: 16.85%; PC3: 8.01%, PC4: 4.81%, and PC5: 1.35%). The prediction correlation coefficients (R^2^), calibration and cross-validation errors (RMSEC and RMSECV), and residual prediction deviation (RPD) for each odorant series in this model are detailed in Table 3 and Supplementary Material, Figure S1.

Table 3 provides a detailed evaluation of the E-nose predictive performance for the 11 OS analyzed. For the “chemical” series, the model achieves moderate predictive accuracy, with an R^2^ of 0.71 in calibration and 0.56 in cross-validation, along with an RPD of 1.51. This indicates an acceptable predictive capability, though some limitations persist. Similarly, the “fruity/ripe fruit” series shows improved performance, achieving an R^2^ of 0.82 in calibration and 0.70 in cross-validation, along with an RPD of 1.84, suggesting a reasonable level of predictive reliability. The “green fruit” series shows excellent predictive capability, with high R^2^ values of 0.94 in calibration and 0.90 in cross-validation, and an RPD of 3.29, indicating strong performance in predicting this specific OS. The “green” series also shows strong predictive performance, with an R^2^ of 0.89 in calibration and 0.83 in cross-validation, and an RPD of 2.49, reflecting good model reliability. Other series, such as “floral” and “creamy”, exhibit moderate predictive accuracy, with RPD values of 1.93 and 2.35, respectively, and R^2^ values that confirm the model’s ability to capture the variability in the data, albeit with some space for improvement. However, the model shows weaker predictive power for certain series like “toasty/smoky” and “waxy”, with lower R^2^ values (0.70 and 0.69 in calibration) and RPD values of 1.52 and 1.54. This suggests that the E-nose has limited effectiveness in predicting the specific volatile compounds associated with these series. Notably, the “honey” series stands out with robust predictive performance, achieving an R^2^ of 0.91 in calibration and 0.85 in cross-validation, along with an RPD of 2.71. RPD values are a critical measure to evaluate the robustness and predictive power of a model, especially in complex multivariate analyses such as those involving E-nose data [46].

Unlike R^2^, which measures how well the model explains the variance in the calibration set, the RPD accounts for the relationship between data variability (as measured by the standard deviation) and the model’s predictive error. This makes the RPD especially important for assessing the practical applicability of the model to real-world data. High RPD values (typically greater than 3) indicate strong predictive capabilities, meaning the model can reliably forecast new data based on learned relationships from the training set. Conversely, lower RPD values (below 2) indicate that the model may struggle to generalize well beyond the calibration data, which limits its practical use [47]. A notable observation in the presented data is the significant decrease in the predictive R^2^ (R^2^ CV) when moving from calibration to cross-validation for some odorant series, such as “chemical” and “citrus”. This decrease indicates that the model is likely overfitted to the calibration data. Overfitting occurs when a model becomes too closely tailored to the specific patterns and noise within the training dataset, capturing not only true underlying relationships but also random fluctuations that do not generalize to new data. As a result, while the model may exhibit high accuracy during calibration (as reflected by high R^2^ Cal values), its performance declines when exposed to unseen data in cross-validation, leading to a lower R^2^ CV. This behavior highlights the importance of balancing model complexity with generalization capability, ensuring the model remains flexible enough to accurately predict new wine samples while avoiding overfitting. Therefore, the combined analysis of RPD and R^2^ provides insight into both the strengths and limitations of the E-nose model, identifying areas where further optimization and validation are needed.

## 3. Materials and Methods

### 3.1. Grape Must and Fermentation Conditions

Grape must from Pedro Ximénez grapes grown in the Montilla-Moriles region (Córdoba, southern Spain), with a density of 1088 g L^−1^ and a pH of 3.78, was added with 100 mg L^−1^ of potassium metabisulfite (K_2_S_2_O_5_) and acidified with solid tartaric acid until it reached pH 3.41. This juice was then pooled into ten 2 L Pyrex graduated cylinders, filled up to 1.75 L, and a starter culture of selected yeast was inoculated to each cylinder for further alcoholic fermentation. This process was carried out at a constant temperature of 20 °C until the end of the process (d ≤ 995 g/L), when the young wine was obtained.

### 3.2. Yeasts and Inoculation Conditions

Five inoculation/fermentation strategies were tested, each in duplicate. The first fermentation, used as a control, was carried out with wild yeasts naturally present on the grapes, without adding starter cultures (hereafter referred to as WY). The other four strategies were carried out with the addition of a starter culture of one active dry yeast (ADY) only. Three yeasts were tested: Glutaferm One^®^, a *Saccharomyces cerevisiae* (SC) strain from AEB (Stuttgart-Möhringen, Germany), improved by adaptative evolution for high glutathione (GSH) production and low production of hydrogen sulfide (H_2_S), and two non-*Saccharomyces* yeasts: Primaflora VB^®^, *M. pulcherrima* (MP), (AEB), and *L. thermotolerans* (LT), (Laktia™, Lallemand^®^, Cornwall, ON, Canada). Free format starter cultures of each yeast were added to their respective cylinders. In addition, *L. thermotolerans* was tested in an immobilized format in microbial biocapsules (hereafter referred to as BC), also in its respective cylinders. The free format is the conventional method used by winemakers, while the immobilized format—using microbial biocapsules containing a non-*Saccharomyces* yeast—was tested for the first time in these fermentations using the E-nose technique.

#### 3.2.1. Preparation of Starter Culture of ADY in Free Format

Each ADY was rehydrated and reactivated following the supplier’s instructions and aliquots of each culture were added to the respective cylinders to obtain a population of 2 × 10^6^ cells mL^−1^.

#### 3.2.2. Preparation of Immobilized *L. thermotolerans* Starter Culture

ADYs of *L. thermotolerans* were pre-cultured as described for the free format. Yeast cells were pelleted (7000 rpm, 15 min) and immobilized into microbial biocapsules, following Muñoz-Castells et al. (2024) [7]. This method combined yeast cells with *Aspergillus oryzae* hyphal pellets, distinguishable if observed under a 40× microscope. A specific weight of biocapsules was inoculated into the grape must to achieve 2 × 10⁶ cells mL⁻^1^. The immobilization procedure is a patent-pending methodology (publication number: WO/2024/068943).

### 3.3. Analytical Methods

The enological parameters ethanol, titratable acidity, volatile acidity, pH, and reducing sugars were quantified following the protocols established by OIV (2021) [48]. Lactic and malic acids were measured using reflectometry with Reflectoquant™ (Merck^®^, Darmstadt, Germany). Glutathione levels were determined via Ultra Performance Liquid Chromatography coupled with tandem mass spectrometry (LC-MS/MS), employing an Acquity H-Class UPLC (Waters, Mildford, MA, USA) and QTRAP 5500 Mass Detector (Sciex, Concord, ON, Canada).

In this work, major and minor volatile compounds were quantified. The analysis of major volatile compounds and polyols in wine was performed using gas chromatography (GC) on an Agilent 6890 (Agilent technologies, Santa Clara, CA, USA) equipped with a flame ionization detector (FID) and a CP-WAX 57 CB capillary column. The methodology has been described in Muñoz-Castells et al. (2024) [13] and briefly consists of adding 1 mL of a 4-methyl-2-pentanol solution (as an internal standard) to 10 mL of the wine sample, followed by the addition of 0.2 g of calcium carbonate. This mixture was stirred in an ultrasonic bath for 30 s and centrifuged at 5000 rpm for 10 min at 2 °C. Then, 0.7 µL of the supernatant was injected into the GC inlet. Calibration curves were made from standard solutions of pure compounds of known concentrations and subjected to the same treatment as the samples for absolute quantification of these compounds.

The quantification of minor volatiles was carried out by the SBSE-TD-GC-MS (Stir Bar Sorptive Extraction–Thermal Desorption–Gas Chromatography–Mass Spectrometry) methodology. The equipment consists of an Agilent 7890A GC (Agilent technologies, Santa Clara, CA, USA), coupled with a MSD 5975C (Wilmington, DE, USA) detector, and a Gerstel Multi-Purpose Sampler (MPS) (GmbH & Co. KG-Mülheim an der Rhur, Germany). ChemStation v. 02.02.1431 (Agilent) and Maestro v. 1.0. (Gerstel) software controlled the system and processed the chromatographic data. The extraction of volatile compounds from the wine was performed by a Twister stir bar, coated with polydimethylsiloxane (PDMS), which adsorbs the low-polarity and apolar compounds. Briefly, 1 mL of wine, 0.1 mL of an internal standard solution (hexyl butyrate), and 8.9 mL of buffered ethanol solution (12%, pH 3.5) were stirred at 1200 rpm and 20 °C for 120 min. After rinsing and drying, the Twister was placed in a desorption tube and transferred via MPS to the Thermal Desorption Unit (TDU) for GC-MS analysis. The volatiles were separated on an HP-5MS capillary column (60 m, 0.25 mm, 0.25 µm) with the oven temperature increasing from 50 °C to 190 °C at 4 °C min^−1^. The mass spectrometer operated in electron impact (EI) mode, scanning a mass range of 35–550 Da. All samples were analyzed in triplicate and volatile quantification was based on calibration curves obtained from standard solutions of pure compounds supplied by Sigma-Aldrich and Merck, which were subjected to the same procedure as the samples.

Identification of both major and minor volatile compounds was confirmed by GC-MS under the same conditions as the samples. Mass spectra were compared with those of the NIST08 and Wiley7 libraries, as well as the NIST Chemistry Web database. Further validation involved subjecting the pure commercial compounds to identical analytical conditions used for the samples.

OAVs were calculated by dividing the concentration of each compound by its OPT. The obtained values constitute a valuable approximation of the contribution of volatiles to the overall wine aroma and to the sensory evaluation, explained thus because this method is commonly used in current research [31,32]. In addition, the use of OS, considered as the grouping of volatiles with the same or similar descriptors [49], constitutes an approximation of the attributes evaluated by tasters in the organoleptic analysis. In this study, volatile compounds were grouped into 11 OS following the classifications outlined by Muñoz-Castells et al. (2024) [7].

### 3.4. Electronic Nose Measurement

The electronic nose (E-nose), designed and developed at the University of Rome Tor- Vergata [50], works with an array of 12 quartz crystal microbalances (QMBs) that detect changes in mass (Δm) on the absorbing layer of their surface, which results in the corresponding frequency shift (Δf) in the output signal of the oscillator circuit. Within a small range of variation, the Δf is directly proportional to Δm. The QMBs are fabricated from AT-cut quartz crystals with a 20 MHz fundamental frequency, which provides a mass resolution down to a few nanograms. The QMBs were functionalized with seven metal complexes (Mg, Co, Cu, Zn, Fe, Mn, and Sn), along with free-base porphyrin (H2) and copper, phosphorus, and manganese complexes of triphenylcorrole (TPC). These molecules were deposited over the quartz surface via spray casting and thoroughly characterized for their sensitivity to volatile organic compounds. Each QMB is connected to an oscillator circuit, with a temperature-compensated quartz crystal used as a reference to measure the oscillator’s output frequencies, providing a frequency resolution of 0.1 Hz. The system also includes temperature and relative humidity sensors. Gas flow is regulated by a tubeless pneumatic system that includes a poly(methyl methacrylate) manifold with two inlets and one outlet. This system is connected to a miniature diaphragm pump (flow range 0–200 sccm), a three-way electronic valve, a proportional electronic valve, and a flow sensor. The device is powered and connected via USB, with data acquisition, instrument control, and settings managed through proprietary software developed in Matlab R2013a v. 8.1 (MathWorks^®^, Natick, MA, USA) [50].

Wine analysis was performed as follows: 10 mL of wine was incubated in 25 mL silicone septum-sealed vials at 30 °C for 20 min. The headspace was then extracted for 90 s with a stream of filtered air and directed into the electronic nose sensor chamber. To reset the reference signal, clean air was passed through the E-nose for 300 s after each measurement. Sensor responses were determined by measuring the resonant frequency shift between two steady states—before and after exposure to the wine sample. The resulting sensor signals produced distinct fingerprints representing the overall headspace composition [14].

### 3.5. Statistical Analysis

Data analysis was conducted using various statistical software packages. Analysis of Variance (ANOVA) and Fisher’s least significant difference (LSD) test were performed using Statgraphics Centurion XVI (v. 16.1.11). E-nose data were auto-scaled and analyzed with partial least squares discriminant analysis (PLS-DA) using Venetian-blind cross-validation (blind thickness = 1). Similarly, OS values data were auto-scaled and analyzed through PCA, also using Venetian-blind cross-validation with the same blind thickness. Pearson correlation analysis was conducted to evaluate relationships between QMB responses and identified OS data. Both datasets were normalized by mean-centering.

PCR was applied to develop a linear regression model, using QMB responses as predictors and OS data as responses. The optimal number of latent variables (LVs) was determined using scree plots to balance observed and latent variables by minimizing calibration (RMSEC) and prediction errors (RMSECV). Additionally, the model performance was assessed with prediction correlation coefficients (R^2^) and the ratio of performance to deviation (RPD).

All multivariate analyses were conducted in Matlab R2013a v. 8.1 (MathWorks^®^, Natick, MA, USA) using the PLS Toolbox v. 8.5.2 (Eigenvector Research, Inc., Manson, WA, USA).

## 4. Conclusions

Fermentation of the same grape must using five strategies, with *Saccharomyces* or non-*Saccharomyces* yeasts, resulted in five different wines, each exhibiting different levels of odor active compounds grouped in 11 OS. The PCA of OS values revealed consistent clustering patterns, highlighting the influence of the fermentation strategy on the wine aroma profile.

Data generated by an E-nose equipped with 12 QMB sensors and subjected to a PLS-DA also provided a reliable classification of the wines.

Both the PCA model based on OS data and the PLS-DA model derived from E-nose data consistently identified similar clustering patterns. This highlights the complementary relationship between volatile compound content grouped by OS and the E-nose’s pattern recognition abilities, which objectively differentiate wines based on aroma.

Predictive models were developed using OS and E-nose data to identify the OS that contribute most to wine aroma. These models demonstrated strong predictive capabilities for certain OS but showed limited performance for others, indicating the need for further refinements. While the model showed high accuracy during calibration, its performance declined when tested with unseen data during cross-validation. In order to validate this methodology on a larger scale, additional experiments with a wider range of wines, such as those made with different grape varieties or yeast strains, are necessary. In addition, further experiments in wineries should be developed to investigate the effect of industrial fermentation volumes as a prerequisite to incorporate this technology in the wine industry.

## Figures and Tables

**Table 1:** Mean and standard deviation of the analyzed enological parameters. Different letters in the same row indicate statistical differences in the normalized and scaled data at the 0.05 level according to Fisher’s least significant difference test. WY: obtained by spontaneous fermentation; SC: using *Saccharomyces cerevisiae* active dry yeast; MP: using *Metschnikowia pulcherrima* active dry yeast; LT: using *L. thermotolerans* yeast in free format.

	WY	SC	MP	LT	BC
Ethanol (% *v*/*v*)	13.95 ± 0.05 ^c^	13.75 ± 0.27 ^c^	14.10 ± 0.01 ^c^	12.30 ± 0.33 ^a^	12.9 ± 0.5 ^b^
pH	3.23 ± 0.01 ^b^	3.29 ± 0.00 ^c^	3.18 ± 0.01 ^a^	3.39 ± 0.03 ^d^	3.40 ± 0.02 ^d^
Volatile acidity (g L^−1^)	0.27 ± 0.01 ^a^	0.46 ± 0.00 ^c^	0.27 ± 0.03 ^a^	0.39 ± 0.00 ^b^	0.44 ± 0.02 ^c^
Total acidity (g L^−1^)	7.70 ± 0.05 ^c^	6.30 ± 0.08 ^a^	7.17 ± 0.04 ^b^	9.5 ± 0.2 ^e^	8.80 ± 0.08 ^d^
Reducing sugars (g L^−1^)	0.17 ± 0.00 ^a^	0.14 ± 0.00 ^a^	0.22 ± 0.00 ^ab^	0.6 ± 0.2 ^b^	2.4 ± 0.7 ^c^
Lactic acid (g L^−1^)	0.23 ± 0.02 ^a^	0.18 ± 0.00 ^a^	0.25 ± 0.01 ^a^	4.7 ± 0.5 ^c^	4.0 ± 0.4 ^b^
Malic acid (g L^−1^)	0.88 ± 0.07 ^bc^	0.84 ± 0.02 ^d^	0.81 ± 0.01 ^cd^	0.44 ± 0.02 ^a^	0.55 ± 0.09 ^ab^
Gluthatione (mg L^−1^)	0.64 ± 0.04 ^b^	1.99 ± 0.19 ^c^	0.25 ± 0.01 ^a^	6.61 ± 0.12 ^e^	3.2 ± 0.7 ^d^

**Table 2:** Mean and standard deviation contents of Odor Activity Values for minor volatile compounds quantified in wines. Different letters in the same row indicate statistical differences in the normalized and scaled data at the 0.05 level according to Fisher’s least significant difference test, denoted in the table as homogeneous groups (HGs). WY: obtained by spontaneous fermentation; SC: using *Saccharomyces cerevisiae* active dry yeast; MP: using *Metschnikowia pulcherrima* active dry yeast; LT: using *Lachancea thermotolerans* yeast in free format; BC: with *L. thermotolerans* immobilized. OS: odorant series. * Indicates an OAV value higher than 0.2.

Compounds	WY	SC	MP	LT	BC	HG	OS
**Acetates (9)**							
Ethyl acetate	7.83 ± 0.09 *^c^	5.7 ± 0.2 *^b^	5.0 ± 0.2 *^a^	11.5 ± 0.9 *^e^	9.4 ± 0.4 *^d^	4	1,2,4
Butyl acetate	0.00034 ± 0.00009 ^a^	0.0002 ± 0.0001 ^a^	0.00035 ± 0.00004 ^a^	0.00081 ± 0.00009 ^b^	0.0008 ± 0.0001 ^b^	2	2
Isoamyl acetate	24 ± 2 *^c^	19 ± 3 *^b^	9.6 ± 0.9 *^a^	11 ± 2 *^a^	18 ± 2 *^b^	3	2
(Z)-3-Hexenyl acetate	0.5 ± 0.2 *^c^	0.75 ± 0.07 *^d^	0.0001 ± 0 ^a^	0.22 ± 0.06 *^b^	0.28 ± 0.03 *^b^	4	2,4
Hexyl acetate	0.7 ± 0.3 *^b^	1.1 ± 0.2 *^c^	0.0005 ± 0 ^a^	0.11 ± 0.02 ^a^	0.15 ± 0.05 ^a^	3	2,3
Octyl acetate	0.115 ± 0.005 ^ab^	0.121 ± 0.007 ^b^	0.112 ± 0.002 ^a^	0.113 ± 0.007 ^ab^	0.12 ± 0.01 ^ab^	2	5,11
Ethyl phenylacetate	0.031 ± 0.004 ^c^	0.009 ± 0.002 ^a^	0.023 ± 0.005 ^b^	0.051 ± 0.004 ^e^	0.041 ± 0.008 ^d^	5	5,10
2-Phenylethyl acetate	15 ± 1 *^d^	3.6 ± 0.2 *^c^	2.4 ± 0.2 *^b^	1.3 ± 0.2 *^a^	2.2 ± 0.1 *^b^	4	5,10
Geranyl acetate	0.30 ± 0.09 *^b^	0.21 ± 0.04*^a^	0.54 ± 0.03 *^d^	0.41 ± 0.03 *^c^	0.45 ± 0.04 *^c^	3	5
**Ethyl esters (13)**							
Ethyl lactate	0.17 ± 0.02 ^a^	0.166 ± 0.007 ^a^	0.21 ± 0.02 *^a^	0.95 ± 0.09 *^c^	0.80 ± 0.07 *^b^	3	2
Ethyl isobutyrate	1.9 ± 0.2 *^c^	0.60 ± 0.09 *^a^	1.08 ± 0.06 *^b^	3.9 ± 0.2 *^e^	3.5 ± 0.3 *^d^	5	2
Ethyl butyrate	1.9 ± 0.2 *^a^	1.9 ± 0.3 *^a^	2.6 ± 0.3 *^b^	1.8 ± 0.2 *^a^	1.7 ± 0.1 *^a^	2	2
Ethyl 2-methylbutyrate	0.0 ± 0.0 ^a^	0.0 ± 0.0 ^a^	0.0 ± 0.0 ^a^	0.0 ± 0.0 ^a^	0.16 ± 0.02 ^b^	2	2
Ethyl 3-methylbutyrate	1.7 ± 0.3 *^b^	0.0 ± 0.0 ^a^	0.0 ± 0.0 ^a^	0.0 ± 0.0 ^a^	0.0 ± 0.0 ^a^	2	2,3
Diethyl succinate	0.118 ± 0.04 ^c^	0.079 ± 0.007 ^b^	0.08 ± 0.02 ^b^	0.0 ± 0.0 ^a^	0.02 ± 0.02 ^a^	3	2
Ethyl hexanoate	0.0001 ± 0.0000 ^a^	0.0001 ± 0.0000 ^a^	0.0001 ± 0.0000 ^a^	0.0001 ± 0.0000 ^a^	0.0001 ± 0.0000 ^a^	1	2,3
Ethyl heptanoate	0.087 ± 0.005 ^d^	0.055 ± 0.005 ^b^	0.0 ± 0.0 ^a^	0.058 ± 0.007 ^bc^	0.062 ± 0.006 ^c^	4	2,3
Ethyl octanoate	0.0002 ± 0.0000 ^a^	0.0002 ± 0.0000 ^a^	0.0002 ± 0.0000 ^a^	0.0002 ± 0.0000 ^a^	0.0002 ± 0.0000 ^a^	1	2,11
Ethyl decanoate	0.113 ± 0.005 ^ab^	0.12 ± 0.02 ^b^	0.114 ± 0.009 ^ab^	0.106 ± 0.004 ^a^	0.15 ± 0.02 ^c^	3	2,11
Ethyl dodecanoate	0.0069 ± 0.0004 ^a^	0.0063 ± 0.0003 ^a^	0.0069 ± 0.0003 ^a^	0.007 ± 0.002 ^a^	0.020 ± 0.003 ^b^	2	11
Ethyl tetradecanoate	0.0042 ± 0.0003 ^b^	0.0041 ± 0.0002 ^b^	0.0042 ± 0.0003 ^b^	0.0034 ± 0.0004 ^a^	0.0042 ± 0.0004 ^b^	2	5,6
Ethyl hexadecanoate	0.0064 ± 0.0008 ^b^	0.0049 ± 0.0005 ^a^	0.008 ± 0.002 ^c^	0.0040 ± 0.0007 ^a^	0.0052 ± 0.0005 ^ab^	3	2,6,11
**Other esters (3)**							
Cis-3-Hexenyl butyrate	9 ± 1 *^c^	7 ± 1 *^a^	8.6 ± 0.8 *^bc^	8 ± 1 *^ab^	7.1 ± 0.8 *^a^	3	4
2-Phenylethyl butanoate	0.004 ± 0.002 ^b^	0.000005 ± 0.000000 ^a^	0.010 ± 0.002 ^c^	0.004 ± 0.002 ^b^	0.009 ± 0.001 ^c^	3	5
E-Methyldihydrojasmonate	0.012 ± 0.007 ^ab^	0.02 ± 0.02 ^b^	0.02 ± 0.01 ^b^	0.008 ± 0.002 ^a^	0.011 ± 0.002 ^ab^	2	5
**Alcohols (10)**							
Methanol	0.062 ± 0.005 ^a^	0.09 ± 0.01 ^b^	0.067 ± 0.004 ^a^	0.060 ± 0.003 ^a^	0.118 ± 0.007 ^c^	3	1
1-Propanol	0.027 ± 0.002 ^b^	0.023 ± 0.001 ^a^	0.029 ± 0.001 ^b^	0.083 ± 0.003 ^c^	0.082 ± 0.005 ^c^	3	1,4
Isobutanol	1.6 ± 0.1 *^b^	0.79 ± 0.02 *^a^	2.34 ± 0.04 *^d^	1.87 ± 0.09 *^c^	1.93 ± 0.08 *^c^	4	1
2-Methyl-1-butanol	1.80 ± 0.07 *^b^	1.56 ± 0.05 *^a^	2.56 ± 0.05 *^d^	2.42 ± 0.06 *^c^	2.7 ± 0.1 *^e^	5	1
3-Methyl-1-butanol	10.5 ± 0.3 *^c^	9.1 ± 0.3 *^a^	10.0 ± 0.1 *^b^	10.6 ± 0.4 *^cd^	10.8 ± 0.3 *^d^	4	1
2-Phenylethanol	6 ± 1 *^ab^	5.0 ± 0.3 *^a^	6.2 ± 0.4 *^b^	8.2 ± 0.7 *^c^	8.7 ± 0.5 *^c^	3	5
Hexanol	0.10 ± 0.01 ^a^	0.092 ± 0.007 ^a^	0.094 ± 0.006 ^a^	0.09 ± 0.01 ^a^	0.118 ± 0.006 ^b^	2	4
2-Ethyl-1-hexanol	0.0017 ± 0.0004 ^a^	0.0021 ± 0.0004 ^ab^	0.0017 ± 0.0002 ^a^	0.0024 ± 0.0006 ^b^	0.0024 ± 0.0004 ^b^	2	7
Dodecanol	0.006 ± 0.003 ^cd^	0.0053 ± 0.0005 ^bc^	0.0074 ± 0.0008 ^d^	0.004 ± 0.001 ^ab^	0.0032 ± 0.0008 ^a^	4	11
2-Methoxy-4-vinylphenol	0.7 ± 0.1 *^b^	0.8 ± 0.2 *^bc^	0.83 ± 0.02 *^c^	0.000008 ± 0.0 ^a^	0.000008 ± 0.0 ^a^	3	9
**Lactones (4)**							
γ-Butyrolactone	0.40 ± 0.07 *^a^	0.48 ± 0.02 *^b^	0.71 ± 0.04 *^c^	0.41 ± 0.05 *^a^	0.39 ± 0.03 *^a^	3	6
γ-Crotonolactone	0.0 ± 0.0 ^a^	0.0 ± 0.0 ^a^	0.0 ± 0.0 ^a^	0.0 ± 0.0 ^a^	0.0 ± 0.0 ^a^	1	6
γ-Nonalactone	0.20 ± 0.03 *^c^	0.17 ± 0.02 ^b^	0.39 ± 0.03 *^d^	0.14 ± 0.03 ^a^	0.21 ± 0.02 *^c^	4	6,2
β-Damascenone	47 ± 2 *^b^	62 ± 9 *^d^	55 ± 4 *^c^	38 ± 3 *^a^	43 ± 5 *^ab^	4	5,8
**Carbonyl compounds (10)**							
Acetaldehyde	7 ± 1 *^ab^	9.5 ± 0.9 *^b^	6.4 ± 0.4 *^a^	20 ± 4 *^c^	21 ± 2 *^c^	3	1,2
1,1-Diethoxyethane	0.0 ± 0.0 ^a^	0.010 ± 0.003 ^a^	0.0 ± 0.0 ^a^	8 ± 2 *^b^	1.45 ± 0.04 *^a^	2	1,4
Acetoin	1.2 ± 0.1 *^a^	0.99 ± 0.06 *^a^	1.06 ± 0.07 *^a^	4.8 ± 0.3 *^b^	5.7 ± 0.6 *^c^	3	6
Hexanal	0.33 ± 0.08 *^ab^	0.24 ± 0.09 *^a^	0.48 ± 0.08 *^c^	0.46 ± 0.07 *^c^	0.39 ± 0.06 *^bc^	3	4
Furfural	0.7 ± 0.2 *^a^	0.6 ± 0.2 *^a^	0.7 ± 0.1 *^a^	0.57 ± 0.04 *^a^	0.9 ± 0.2 *^b^	2	1,9
Benzaldehyde	0.0 ± 0.0 ^a^	0.002 ± 0.002 ^b^	0.0 ± 0.0 ^a^	0.003 ± 0.000 ^b^	0.007 ± 0.002 ^c^	3	2
Octanal	0.0 ± 0.0 ^a^	0.33 ± 0.02 *^b^	0.4 ± 0.1 *^bc^	0.6 ± 0.1 *^d^	0.4 ± 0.1 *^c^	4	7
Nonanal	2.9 ± 0.3 *^a^	3.3 ± 0.3 *^b^	3.6 ± 0.3 *^bc^	3.7 ± 0.2 *^c^	3.4 ± 0.2 *^b^	3	7
2-Phenylacetaldehyde	0.0 ± 0.0 ^a^	0.0 ± 0.0 ^a^	0.0 ± 0.0 ^a^	8 ± 1 *^b^	10.3 ± 0.7 *^c^	3	4,10
Decanal	4.4 ± 0.4 *^a^	5.2 ± 0.2 *^b^	5.0 ± 0.6 *^b^	6.3 ± 0.5 *^c^	6.1 ± 0.2 *^c^	3	8,11
**Terpenes and derivatives (5)**							
Limonene	468 ± 27 *^b^	348 ± 30 *^a^	322 ± 44 *^a^	352 ± 20 *^a^	320 ± 34 *^a^	2	1,7
E-Geranyl acetone	0.024 ± 0.008 ^d^	0.017 ± 0.003 ^bc^	0.022 ± 0.005 ^cd^	0.011 ± 0.002 ^a^	0.015 ± 0.003 ^ab^	4	5
Z-Geranyl acetone	0.0302 ± 0.0009 ^a^	0.030 ± 0.002 ^a^	0.031 ± 0.001 ^a^	0.030 ± 0.002 ^a^	0.0299 ± 0.0009 ^a^	1	5
Nerolidol	0.0 ± 0.0 ^a^	0.0062 ± 0.0004 ^c^	0.002 ± 0.002 ^b^	0.0001 ± 0.0001 ^a^	0.0 ± 0.0 ^a^	3	4,5
Farnesol	0.0001 ± 0.0000 ^a^	0.0001 ± 0.0000 ^a^	0.0001 ± 0.0000 ^a^	0.0001 ± 0.0000 ^a^	0.0001 ± 0.0000 ^a^	1	5
**Miscellaneous (3)**							
2,3-Butanediol *levo*	0.7 ± 0.2 *^b^	0.57 ± 0.05 *^ab^	0.69 ± 0.08 *^b^	0.49 ± 0.05 ^a^	0.47 ± 0.08 *^a^	2	2,6
2,3-Butanediol *meso*	0.3 ± 0.1 *^b^	0.17 ± 0.01 ^a^	0.25 ± 0.02 *^b^	0.19 ± 0.01 ^a^	0.19 ± 0.02 ^a^	2	2,6
2-Pentylfuran	1.0 ± 0.2 *^c^	0.5 ± 0.2 *^b^	0.66 ± 0.06 *^b^	0.0002 ± 0.0000 ^a^	0.0002 ± 0.0000 ^a^	3	3

OS: 1: chemical; 2: fruity/ripe fruit; 3: green fruit; 4: green; 5: floral; 6: creamy; 7: citrus; 8: herbaceous; 9: toasty/smoky; 10: honey; and 11: waxy.

**Table 3:** Statistical results of the principal component regression model with five latent variables and N = 30. The mean of 30 replicates with the minimum (Min) and maximum (Max) values as well as standard deviation for each of the odorant series are reported. RMSEC, root mean square error in calibration; RMSECV, root mean square error in cross-validation; R_2_ Cal, correlation coefficient in calibration; R_2_ CV, correlation coefficient in cross-validation; RPD, residual prediction deviation (SD/RMSEC CV).

Odorant Series	Mean	Min	Max	SD	RMSEC	RMSEC CV	R^2^ Cal	R^2^ CV	RPD
Chemical	389.43	291.34	522.09	60.67	31.89	40.12	0.71	0.56	1.51
Fruity/ripe fruit	41.26	28.66	53.35	7.17	2.92	3.9	0.82	0.70	1.84
Green fruit	1.23	0.14	3.7	1.25	0.3	0.38	0.94	0.90	3.29
Green	22.31	12.76	37.35	9.22	2.91	3.71	0.89	0.83	2.49
Floral	61.18	42.85	84.94	10.13	4.27	5.25	0.81	0.72	1.93
Creamy	4.26	2.29	7.74	1.93	0.66	0.82	0.87	0.81	2.35
Citrus	365.81	263	507.1	62.63	32.56	41.36	0.72	0.56	1.51
Herbaceous	54.34	38.1	81.87	9.78	4.43	5.38	0.78	0.69	1.82
Toasty/smoky	1.15	0.52	1.84	0.41	0.22	0.27	0.70	0.53	1.52
Honey	8.61	2.24	16.64	4.99	1.42	1.84	0.91	0.85	2.71
Waxy	5.66	4.09	7.36	0.83	0.44	0.54	0.69	0.55	1.54

## Data Availability

The data presented in this study are available upon request from the corresponding author.

## Supplementary Materials

The following supporting information can be downloaded at: https://www.mdpi.com/article/10.3390/molecules30071584/s1. Figure S1: Scatter plots referred to the PCR model (observed vs. predicted data) based on the e-nose sensors signal, for the following odorant series: Chemical, Fruity/ripe fruit, Green fruit, Green, Floral, Creamy, Citrus, Herbaceous, Toasty/smoky, Honey, Waxy. Table S1: Quantified compounds in wines. Table S2: Odorant series identified in the different wines. Table S3: Confusion matrix of the PLS-DA shown in Figure 2. Refs. [51,52,53,54,55,56,57,58,59,60] are cited in Supplementary Materials.

## Abbreviations

The following abbreviations are used in this manuscript:

ADY	Active dry yeast
ANOVA	Analysis of Variance
BC	Biocapsules of *Lachancea thermotolerans*
CV	Cross-validation
E-nose	Electronic nose
EI	Electron impact
*f*	Frequency
FID	Flame ionization detector
GC	Gas chromatography
GSH	Glutathione
HG	Homogeneous groups
LSD	Least significant difference
LT	*Lachancea thermotolerans*
LV	Latent variable
*m*	Mass
MP	*Metschnikowia pulcherrima*
MPS	Multi-Purpose Sampler
MS	Mass spectrometry
OAV	Odor Activity Value
OPT	Odor perception threshold
OS	Odorant series
PCA	Principal Component Analysis
PCR	Principal component regression
PDMS	Polydimethylsiloxane
PLS-DA	Partial least squares discriminant analysis
QMB	Quartz crystal microbalances
RMSEC	Root mean square error in calibration
RMSECV	Root mean square error in cross-validation
RPD	Residual prediction deviation
SBSE	Stir Bar Sorptive Extraction
SC	*Saccharomyces cerevisiae*
TD	Thermal Desorption
TDU	Thermal Desorption Unit
TPC	Triphenylcorrole
VIP	Variables of importance in projection
WY	Wild yeast (spontaneous fermentation procedure)
