# Supplementary material for: Application of an Electronic Nose to the Prediction of Odorant Series in Wines Obtained with Saccharomyces or Non-Saccharomyces Yeast Strains
Source: Molecules. 2025 Apr 2;30(7):1584. doi: 10.3390/molecules30071584 (PMC11990477; doi:10.3390/molecules30071584)
Supplement: Supplementary file 1 [file molecules-30-01584-s001.zip › Table S1.pdf]

molecules

MDPI

Supplementary Material

Table S1. Quantified compounds in wines. CAS: identification number assigned by the Chemical Abstracts Service. OPT: odor perception threshold in μg L^{-1} except major volatile compounds in mg L^{-1} (ethyl acetate, Ethyl lactate, Diethyl succinate; Methanol, 1-Propanol, Isobutanol, 2-Methyl-1-butanol, 3-Methyl-1-butanol, 2-Phenylethanol, Acetaldehyde, 1,1-Diethoxyethane, Acetoin, 2-Phenylacetaldehyde, 2,3-Butanediol *levo*, 2,3-Butanediol *meso*). OS: odorant series.

Compounds	CAS	OPT	Aroma descriptor	OS
**Acetates (9)**				
Ethyl acetate	141-78-6	7.5[1]	Ethereal, fruity, sweet, green	1,2,4
Butyl acetate	123-86-4	4600[I]	Sweet, fruity, banana	2
Isoamyl acetate	123-92-2	30[II]	Banana	2
(Z)-3-Hexenyl acetate	3681-71-8	8[III]	Green, apple, pear, melon	2,4
Hexyl acetate	142-92-7	2[II]	Apple, pear	2,3
Octyl acetate	112-14-1	20[IV]	Green, herbal, waxy	5,11
Ethyl phenylacetate	101-97-3	73[V]	Floral, honey, rose	5, 10
2-Phenylethyl acetate	103-45-7	250[II]	Fruity, floral, rose	5, 10
Geranyl acetate	105-87-3	9[VI]	Floral, rose, waxy	5
**Ethyl esters (13)**				
Ethyl lactate	97-64-3	100[1]	Fruity, buttery	2
Ethyl isobutyrate	97-62-1	15[II]	Apple, strawberry	2
Ethyl butyrate	105-54-4	20[II]	Fruity, tutti frutti	2
Ethyl 2-methylbutyrate	7452-79-1	18[II]	Fruity, estery, berry	2
Ethyl 3-methylbutyrate	108-64-5	3[II]	Green pineapple	2,3
Diethyl succinate	123-25-7	100[1]	Fruity, apple	2
Ethyl hexanoate	123-66-0	14[II]	Pineapple, green banana	2,3
Ethyl heptanoate	106-30-9	2.2[II]	Fruity pineapple	2.3
Ethyl octanoate	106-32-1	5[II]	Pineapple, floral	2,11
Ethyl decanoate	110-38-3	200[II]	Fruity, sweet apple, grape	2,11
Ethyl dodecanoate	106-33-2	2000[II]	Creamy, floral	11
Ethyl tetradecanoate	124-06-1	2000[II]	Creamy, waxy, violet	5,6
Ethyl hexadecanoate	628-97-7	2000[II]	Fruity, creamy, milky	2,6,11
**Other esters (3)**				
Cis-3-Hexenyl butyrate	16491-36-4	0.50[VIII]	Green, apple, fruity	4
2-Phenylethyl butanoate	103-52-6	200[II]	Floral, musty	5
E-Methyldihydrojasmonate	24851-98-7	70[IX]	Floral, oily, jasmin	5
**Higher alcohols (10)**				
Methanol	67-56-1	668[1]	Alcohol, Chemical. medicinal	1
1-Propanol	71-23-8	830[1]	Alcoholic, fusel, musty	1,4
Isobutanol	78-83-1	40[1]	Ethereal, fusel alcohol	1
2-Methyl-1-butanol	137-32-6	30[1]	Alcoholic, nail polish	1
3-Methyl-1-butanol	123-51-3	30[1]	Alcohol, nail polish	1

2-Phenylethanol	60-12-8	10[1]	Floral, rose	5
Hexanol	111-27-3	2500[II]	Grass	4
2-Ethyl-1-hexanol	104-76-7	8000[VII]	Citrus, fresh, floral, oily, sweet	7
Dodecanol	112-53-8	1000[IX]	Waxy, soapy, fatty	11
2-Methoxy-4-vinylphenol	7786-61-0	125.8[IX]	spicy, clove, smoky	9
**Lactones (4)**				
γ-Butyrolactone	96-48-0	35000[IX]	Creamy, oily, fatty	6
γ-Crotonolactone	497-23-4	1000[II]	Buttery, toasty	6
γ-Nonalactone	104-61-0	30[II]	Creamy. Coconut	6,2
β-Damascenone	23696-85-7	0.05[III]	Floral, sweet, fruity	5,8
**Carbonyl compounds (10)**				
Acetaldehyde	75-07-0	10[1]	Ethereal, aldehydic, fruity	1,2
1,1-Diethoxyethane	105-57-7	1[1]	Green fruit, liquorice, ethereal	1,4
Acetoin	513-86-0	30[1]	Buttery, creamy, milky, fatty	6
Hexanal	66-25-1	9.1[VII]	Green, fatty, leafy	4
Furfural	98-01-1	770[II]	Burned almonds, fusel alcohol	1,9
Benzaldehyde	100-52-7	1100[II]	Caramel	2
Octanal	124-13-0	2.5[II]	Citrus	7
Nonanal	124-19-6	2.5[II]	Citrus	7
2-Phenylacetaldehyde	122-78-1	1[X]	Honey, floral, rose	4, 10
Decanal	112-31-2	1.25[II]	Citrus	8,11
**Terpenes and derivatives (5)**				
Limonene	5989-27-5	10[II]	Citrus, herbal	1,7
E-Geranyl acetone	689-67-8	60[V]	Floral, rose, leaf	5
Z-Geranyl acetone	689-67-8	60[V]	Floral, rose, leaf	5
Nerolidol	7212-44-4	700[IX]	Floral, green, citrus	4,5
Farnesol	4602-84-0	20[V]	Floral, sweet	5
**Miscelaneous (3)**				
2,3-Butanediol *levo*	24347-58-8	668[1]	Fruity, creamy, buttery	2,6
2,3-Butanediol *meso*	5341-95-7	668[1]	Fruity, creamy, buttery	2,6
2-Pentylfuran	3777-69-3	6[X]	Fruity, green	3

I: (Ogawa et al., 2022); II: (López de Lerma et al., 2018); III: (Zhang et al., 2019); IV: (Cometto-Muñiz et al., 2008); V: (Zhu et al., 2019); VI: (Pardo et al., 2015); VII: (Zhang et al., 2015); VIII: (Song et al., 2023); IX: (Martín-García et al., 2023); X: (Welke et al., 2014). 1: chemical; 2: fruity/ripe fruit; 3: green fruit; 4: green; 5: floral; 6: creamy; 7: citrus; 8: herbaceous; 9: toasty/smoky; 10: honey; 11: waxy.

References

Cometto-Muñiz, J.E.; Cain, W.S.; Abraham, M.H.; Gil-Lostes, J. Concentration-detection functions for the odor of homologous n-acetate esters. *Physiol. Behav.* **2008**, *95*, 658–667. https://doi.org/10.1016/j.physbeh.2008.09.021.

López de Lerma, N.; Peinado, R.A.; Puig-Pujol, A.; Mauricio, J.C.; Moreno, J.; García-Martínez, T. Influence of two yeast strains in free, bioimmobilized or immobilized with alginate forms on the aromatic profile of long aged sparkling wines. *Food Chem.* **2018**, *250*, 22–29. https://doi.org/10.1016/j.foodchem.2018.01.036.

Martín-García, F.J.; Palacios-Fernández, S.; López de Lerma, N.; García-Martínez, T.; Mauricio, J.C.; Peinado, R.A. The effect of yeast, sugar and sulfur dioxide on the volatile compounds in wine. *Fermentation* **2023**, *9*, 541. https://doi.org/10.3390/fermentation9060541.

Ogawa, M.; Vararu, F.; Moreno-Garcia, J.; Mauricio, J.C.; Moreno, J.; Garcia-Martinez, T. Analyzing the minor volatilome of *Torulaspora delbrueckii* in an alcoholic fermentation. *Eur. Food Res. Technol.* **2022**, *248*, 613–624. https://doi.org/10.1007/s00217-021-03910-y.

Pardo, E.; Rico, J.; Gil, J.V.; Orejas, M. *De novo* production of six key grape aroma monoterpenes by a geraniol synthase-engineered *Saccharomyces cerevisiae* wine strain. *Microb. Cell Fact.* **2015**, *14*, 136. https://doi.org/10.1186/s12934-015-0306-5.

Song, X.; Dai, F.; Yao, J.; Li, Z.; Huang, Z.; Liu, H.; Zhu, Z. Characterization of the volatile profile of *Feijoa* (*Acca sellowiana*) fruit at different ripening stages by HS-SPME-GC/MS. *LWT–Food Sci. Technol.* **2023**, *184*, 115011. https://doi.org/10.1016/j.lwt.2023.115011.

Welke, J.E.; Zanus, M.; Lazzarotto, M.; Alcaraz Zini, C. Quantitative analysis of headspace volatile compounds using comprehensive two-dimensional gas chromatography and their contribution to the aroma of Chardonnay wine. *Food Res. Int.* **2014**, *59*, 85–99. https://doi.org/10.1016/j.foodres.2014.02.002.

Zhang, S.; Petersen, M.A.; Liu, J.; Toldam-Andersen, T.B.; Ebeler, S.E.; Hopfer, H. Influence of pre-fermentation treatments on wine volatile and sensory profile of the new disease-tolerant cultivar Solaris. *Molecules* **2015**, *20*, 21609–21625. https://doi.org/10.3390/molecules201219791.

Zhang, Y.S.; Du, G.; Gao, Y.T.; Wang, L.W.; Meng, D.; Li, B.J.; Brennan, C.; Wang, M.Y.; Zhao, H.; Wang, S.Y.; et al. The effect of carbonic maceration during winemaking on the color, aroma and sensory properties of 'Muscat Hamburg' wine. *Molecules* **2019**, *24*, 3120. https://doi.org/10.3390/molecules24173120.

Zhu, L.X.; Zhang, M.M.; Shi, Y.; Duan, C.Q. Evolution of the aromatic profile of traditional Msalais wine during industrial production. *Int. J. Food Prop.* **2019**, *22*, 911–924. https://doi.org/10.1080/10942912.2019.1612428.
