# Supplementary material for: Application of an Electronic Nose to the Prediction of Odorant Series in Wines Obtained with Saccharomyces or Non-Saccharomyces Yeast Strains
Source: Molecules. 2025 Apr 2;30(7):1584. doi: 10.3390/molecules30071584 (PMC11990477; doi:10.3390/molecules30071584)
Supplement: Supplementary file 1 [file molecules-30-01584-s001.zip › Table S2.pdf]

molecules

MDPI

Supplementary Material

Table S2. Odorant series identified in the different wines. Values are the mean of six replicates ± standard deviation.

	WY	SC	MP	LT	BC
**Chemical**	487 ± 27	366 ± 30	340 ± 44	397 ± 17	358 ± 34
**Fruity/ripe fruit**	50 ± 2	40 ± 3	30 ± 1	41 ± 2	46 ± 3
**Green fruit**	3.4 ± 0.2	1.7 ± 0.3	0.7 ± 0.1	0.17 ± 0.02	0.20 ± 0.05
**Green**	18 ± 2	14 ± 1	14.2 ± 0.9	36.4 ± 0.9	29 ± 1
**Floral**	68 ± 3	71 ± 9	64 ± 4	48 ± 3	54 ± 4
**Creamy**	2.8 ± 0.3	2.4 ± 0.1	3.1 ± 0.2	6.1 ± 0.3	7.0 ± 0.5
**Citrus**	471 ± 27	352 ± 30	326 ± 44	356 ± 20	324 ± 34
**Herbaceous**	52 ± 2	67 ± 9	60 ± 5	44 ± 4	49 ± 5
**Toasty/smoky**	1.4 ± 0.3	1.4 ± 0.3	1.5 ± 0.1	0.56 ± 0.04	0.9 ± 0.2
**Honey**	15 ± 1	3.6 ± 0.2	2.5 ± 0.2	9.6 ± 0.9	12.5 ± 0.7
**Waxy**	4.6 ± 0.4	5.5 ± 0.2	5.2 ± 0.6	6.5 ± 0.5	6.4 ± 0.2
