# Supplementary material for: Application of an Electronic Nose to the Prediction of Odorant Series in Wines Obtained with Saccharomyces or Non-Saccharomyces Yeast Strains
Source: Molecules. 2025 Apr 2;30(7):1584. doi: 10.3390/molecules30071584 (PMC11990477; doi:10.3390/molecules30071584)
Supplement: Supplementary file 1 [file molecules-30-01584-s001.zip › Table S3.pdf]

molecules

MDPI

Supplementary Material

Table S1. Confusion matrix of the PLS-DA shown in figure 2. TPR = true positive ratio; FPR=false positive ratio; TNR= true negative ratio; FNR=false negative ratio; N = number of classes; Err= total error; P= Precision: total positive (TP)/total positive + false positive; F1= F1-score.

**Confusion matrix**	**N**	**TPR**	**FPR**	**TNR**	**FNR**	**Err**	**P**	**F1**
WY	6	1	0	1	0	0	1	1
SC	6	1	0	1	0	0	1	1
MP	6	1	0	1	0	0	1	1
LT	6	1	0	1	0	0	1	1
BC	6	1	0	1	0	0	1	1
**Confusion matrix (CV)**	**N**	**TPR**	**FPR**	**TNR**	**FNR**	**Err**	**P**	**F1**
WY	6	1	0	1	0	0	1	1
SC	6	0.33	0.41	0.95	0.66	0.16	0.66	0.44
MP	6	1	0	1	0	0	1	1
LT	6	0.66	0.08	0.91	0.33	0.13	0.66	0.66
BC	6	0.50	0.25	0.75	0.50	0.30	0.33	0.40
