# Supplementary figures and images for: Application of an Electronic Nose to the Prediction of Odorant Series in Wines Obtained with Saccharomyces or Non-Saccharomyces Yeast Strains
Source: Molecules. 2025 Apr 2;30(7):1584. doi: 10.3390/molecules30071584 (PMC11990477; doi:10.3390/molecules30071584)

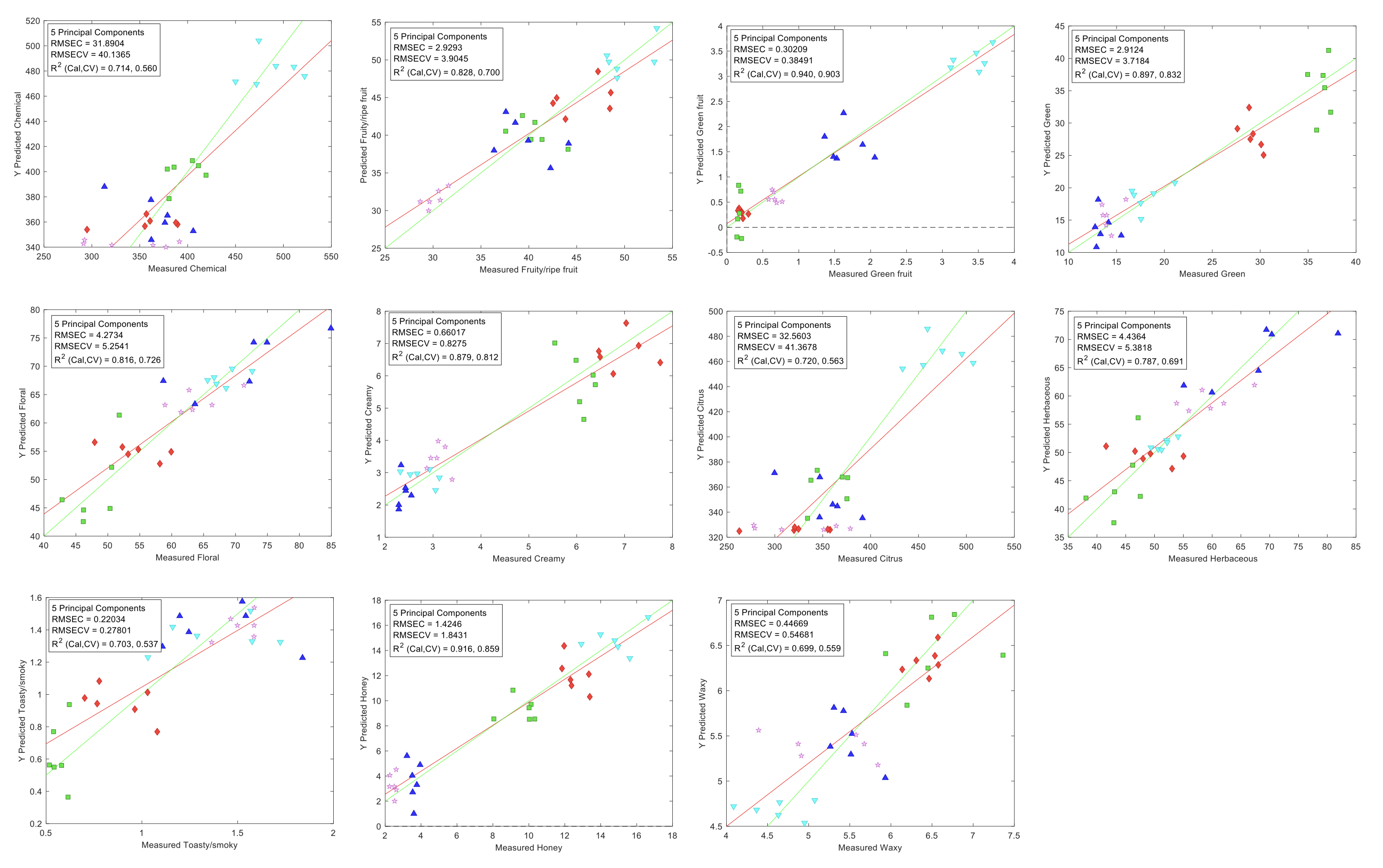

Supplement: Supplementary file 1 [file molecules-30-01584-s001.zip › Figure S1.tiff]
